# Oral lichen planus and other confounding factors in narrow band imaging (NBI) during routine inspection of oral cavity for early detection of oral squamous cell carcinoma: a retrospective pilot study

**DOI:** 10.1186/s12903-019-0762-0

**Published:** 2019-04-30

**Authors:** Agostino Guida, Mariagrazia Maglione, Anna Crispo, Francesco Perri, Salvatore Villano, Ettore Pavone, Corrado Aversa, Francesco Longo, Florinda Feroce, Gerardo Botti, Franco Ionna

**Affiliations:** 1 Maxillofacial and ENT Surgery Department, Istituto Nazionale Tumori – IRCCS “Fondazione G. Pascale”, via M. Semmola, Naples, Italy; 2 Epidemiology and Biostatistics Unit, Istituto Nazionale Tumori – IRCCS “Fondazione G. Pascale”, via M. Semmola, Naples, Italy; 3 Head & Neck Medical Oncology Unit, Istituto Nazionale Tumori – IRCCS “Fondazione G. Pascale”, via M. Semmola, Naples, Italy; 4 Pathological Anatomy and Cytopathology Unit, Istituto Nazionale Tumori – IRCCS “Fondazione G. Pascale”, via M. Semmola, Naples, Italy

**Keywords:** Narrow band imaging, Oral squamous cell carcinoma, Oral potentially malignant disease, Early diagnosis, Follow-up, Oral lichen planus

## Abstract

**Background:**

Narrow Band Imaging is a noninvasive optical diagnostic tool. It allows the visualization of sub-mucosal vasculature; four patterns of shapes of submucosal capillaries can be recognized, increasingly associated with neoplastic transformation. With such characteristics, it has showed high effectiveness for detection of Oral Squamous Cell Carcinoma. Still, scientific literature highlights several bias/confounding factors, such as Oral Lichen Planus. We performed a retrospective observational study on patients routinely examined with Narrow Band Imaging, investigating for bias, confounding factors and conditions that may limit its applicability.

**Methods:**

Age, sex, smoking, use of dentures, history of head & neck radiotherapy, history of Oral Squamous Cell Carcinoma, site of the lesion and thickness of the epithelium of origin were statistically evaluated as possible bias/confounding factors. Pearson’s Chi-squared test, multivariate logistic regression, Positive Predictive Value, Negative Predictive Value, Sensitivity, Specificity, Positive Likelihood Ratio, Negative Likelihood Ratio and accuracy were calculated, normalizing the cohort with/without patients affected by Oral Lichen Planus, to acknowledge its role as bias/confounding factor.

**Results:**

Five hundred fifty-six inspections were performed on 106 oral cavity lesions from 98 patients. Age, sex, smoking, use of dentures and anamnesis of Oral Squamous Cell Carcinoma were not found to influence Narrow Band Imaging. History of head & neck radiotherapy was not assessed due to insufficient sample. Epithelium thickness does not seem to interfere with feasibility. Presence of Oral Lichen Planus patients in the cohort led to false positives but not to false negatives. Among capillary patterns, number IV was the most significantly associated to Oral Squamous Cell Carcinoma (*p* < 0.001), not impaired by the presence of Oral Lichen Planus patients in the cohort (accuracy: 94.3, 95% confidence interval: 88.1–97.9%; odds ratio: 261.7, 95% confidence interval: 37.7–1815.5).

**Conclusion:**

Narrow Band Imaging showed high reliability in detection of Oral Squamous Cell Carcinoma in a cohort of patients with oral cavity lesions not normalized for bias/confounding factors. Still, Oral Lichen Planus may lead to false positives. Narrow Band Imaging could help in the follow-up of patients with multiple lesions through detection of capillary pattern IV, which seems to be the most significantly associated to neoplastic epithelium.

## Background

**Fig. 1:**
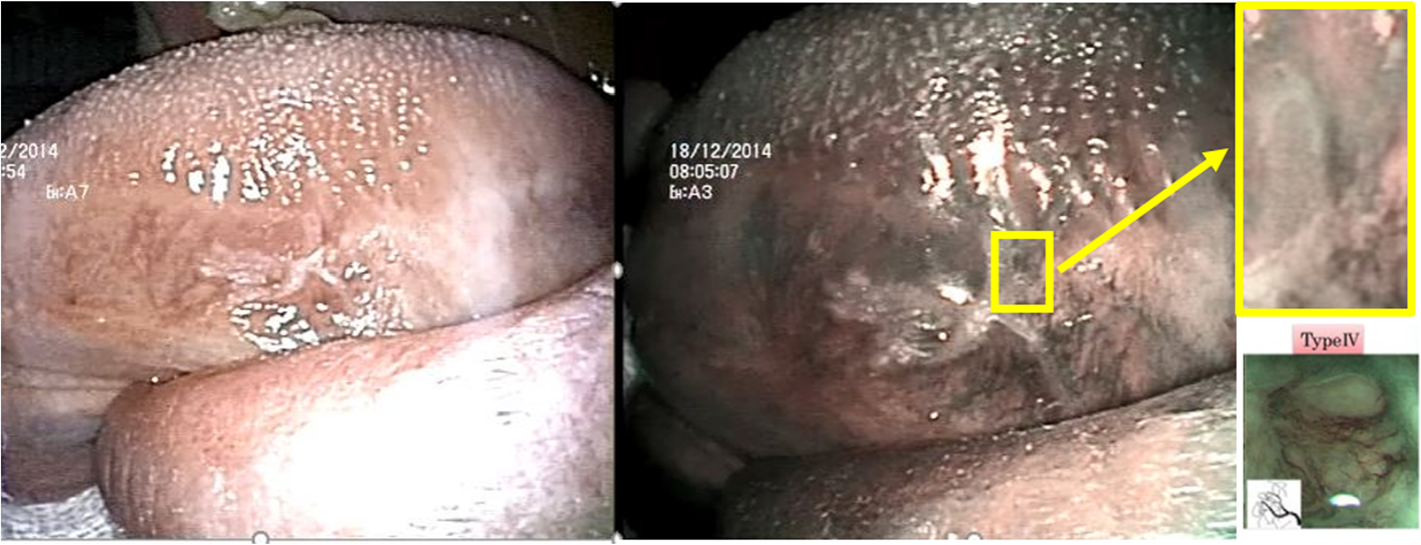
Example of detection of OSCC through NBI. This lesion (not from a patient from the present study), a homogenous leukoplakia at BWL (left image), showed anomalous vascularization at NBI visualization (highlighted spot, middle image). According to IPCL classification criteria, it was classified as an IPCL pattern IV (upper and lower [2] - right images) and the lesion revealed itself a Cis at histopathological examination

Narrow Band Imaging (NBI), a recently introduced noninvasive optical diagnostic technique, allows the visualization of the capillary patterns of the superficial sub-mucosal layer. This device uses narrowed band width filters in a red/green/blue light illumination sequence [1, 2], with wavelengths values for each band being 415 nm and 540 nm. The 415 nm light (blue) highlights the microvasculature [3] among epithelial papillae, providing images of these subtle capillary vessels. NBI, by allowing enhanced inspection of shallow vascular structures of the superficial mucosal layers, has shown its effectiveness for detection of dysplastic/cancerous lesions of the upper aero-digestive tract: oral cavity, oro- and hypopharynx, larynx, esophagus [1–8]. In a recent meta-analysis [9] assessing NBI capability to detect head & neck squamous cell carcinoma (HNSCC), the calculated specificity, sensitivity, negative likelihood rate, positive likelihood rate, and diagnostic odds ratio (OR) of NBI were 95.6, 88.5%, 0.11, 12.33, and 121.26, respectively. Different systems for classifying patterns of intraepithelial capillary vessels (intraepithelial papillary capillary loop, IPCL) have been identified, related to increasing grades of dysplasia/neoplasia of oral cavity [1–4, 6, 8]. In a recent systematic review [10], the four pattern system [2] is considered the most effective in oral cavity, being the all types: type I (physiological arborization of IPCL), type II (circuitous or dilated IPCL), type III (convoluted/winding and/or elongated IPCL) and type IV (complete loss of organization/annihilation of IPCL). A more recent classification has been proposed for oral cavity [11], but it cannot be taken into account as the authors do not show any statistical analysis to corroborate their results. Data from the literature have shown that oral lesions with NBI pattern III and IV have increased chances of anatomopathological diagnosis of High Grade Dysplasia (HGD), oral SCC (OSCC) and OSCC in situ (Cis), when compared to those with the NBI pattern I and II [10, 12, 13]. Furthermore, NBI inspection showed more effective results in predicting HGD/Cis/OSCC (these lesions are usually grouped together in NBI studies on oral mucosa) if compared to use of regular broad-band white light (BWL) and traditional clinical criteria (Fig. 1); the same authors suggested that best results were achieved combining NBI with BWL [12, 13]. NBI has thus found its strong rationale in the follow-up of OSCC patients [14] and in the evaluation of resection margins [15].

Despite these excellent results, routine clinical use in the inspection of oral cavity may present some difficulties. Many studies often exclude Oral Potentially Malignant Disorders (OPMDs), as they are considered confounding factors [9], especially in case of patients with Oral Lichen Planus (OLP) [9–11] due to the dishomogeneous/ulcerated aspect of the lesions and the correlation with high-grade NBI patterns. The site of the lesion is sometimes considered as bias too, due to different types of epithelium found in oral cavity; usually, epithelium of the oral cavity is classified into four categories, namely: lining epithelium (non-keratinized; e.g.: cheek and floor of mouth), masticatory epithelium (keratinized; e.g.: gum), tongue epithelium (keratinized epithelium of the lateral borders of the tongue) and specialized epithelium (on the dorsal tongue) [16]. It has been hypothesized that thicker (e.g.: tongue and masticatory) types of epithelium may obstruct IPCL visualization in the submucosa, but literature is not concordant on this aspect [10, 11]. Furthermore, history of head & neck radiotherapy has been investigated to understand a possible alteration of NBI visualization of the larynx/pharynx [17], but there are no data about oral cavity; the same happens for smoking habits.

On the basis of the aforementioned findings, we performed a retrospective observational study on patients routinely examined with NBI, in order to understand efficacy of NBI in routine oral inspections, investigating also the possible confounding factors and conditions that may limit its applicability.

## Material and methods

This retrospective study was approved by Institutional Scientific Committee and accepted by Ethical Committee (n° DSC-1398/18) of National Tumor Institute INT-IRCCS “Fondazione G. Pascale” (Naples, Italy). From November 2014 to July 2018, all patients necessitating oral cavity inspection presenting at the Outpatient Clinic of Maxillofacial-ENT Surgery Unit of National Tumor Institute INT-IRCCS “Fondazione G. Pascale” (Naples, Italy) underwent intraoral flexible endoscopy with BWL and NBI. Patients’ anamnesis and data were routinely carefully recorded. The examinations were conducted using ENF type VQ flexible endoscope plugged to a CLV-S40Pro light source and a OTV-S7Pro central video system (all items by VISERA Pro – Olympus Medical Systems Corp., Tokyo, Japan). Switch from BWL to NBI view was possible through pressing a button on the flexible endoscope. Oral inspections were first conducted using BWL illumination, for a wide and complete view of all oral mucosa, searching for areas with suspect/pathological clinical appearance, both trough the flexible endoscope and direct observation. The same procedure was followed for NBI examinations, carefully inspecting all of oral mucosa; imaging analysis of the IPCLs characteristics at NBI view was performed by analyzing the intraepithelial capillary vessels patterns of oral mucosa. Images and videos were acquired with a digital recorder (Medicapture Medicap USB200) and stored in a hard drive for eventual further analysis. All NBI oral inspections and pattern assessment were performed by the same operator (AG). When a lesion/area showed different IPCL patterns at NBI, the most advanced one was considered. Incisional/excisional biopsies were performed according to BWL appearance of the lesions or when a NBI pattern III and IV was detected (even without particular BWL clinical suspect/pathological appearance). Biopsies were performed in general or local anesthesia after obtaining informed consent. Histopathological examinations was considered as the diagnostic gold standard and they were performed on paraffin-embedded specimens by the Anatomical Pathology Unit of Our Institute, by a single dedicated pathologist, blinded to the NBI appearance of the lesion; diagnoses were obtained with every necessary coloration and immunohistochemical analysis as per World Health Organization (WHO) 2017 standards [18] for OPMDs and OSCC; specimens analyzed before 2017 were re-evaluated according to the new criteria. The frequency of visits was set for each patient according to their condition/anamnesis and National Cancer Comprehensive Network (NCCN) guidelines.

Patients with lesions appearing white, non-ulcerated, homogeneous, not augmented in consistency, not surrounded by erythematous areas nor protruding from surrounding tissues, with NBI pattern I and II, were considered as “frictional keratosis” (FK - comprehending morsicatio, frictional lesions) [19]. When it was possible to remove causative factors (e.g.: selective grinding of sharp edges of neighboring teeth or prosthetic sharp edges), the patient was re-evaluated in two-weeks; if the lesion did not disappear, biopsy was performed after informed consent was administered to the patient. If the anatomopathological examination confirmed absence of dysplasia/neoplasia, the lesion was then classified as FK. When it was not possible to remove causative factors (e.g. food impaction, bruxism in patient refusing to wear mouth guard), patients were followed-up regularly, on a two-week interval for the first 3 months. These patients were excluded without at least 24 months follow-up.

### Statistical analysis

All statistical analyses were performed with the Statistical Package for Social Science (SPSS) statistical software version 23 (SPSS inc., Chicago IL, USA). In every statistical test performed, *P*-values less than 0.05–95% confidence interval (CI) - were considered significant. Pearson’s Chi-squared test of independence (2-tailed) was used to assess the relationship between categorical variables; sex, age, smoking, use of dentures, history of OSCC, history of Head & Neck radiotherapy, site of the lesion, type of epithelium from which the tumor arose and type of lesion were statistically evaluated to understand their role in influencing NBI patterns. Similarly, relationship between use of dentures and FK presence was assessed. NBI reliability in detecting HGD/Cis/OSCC, using the histopathologic findings as the final diagnostic gold standard to set the status of “true” or “false” positive and negative, was thus evaluated through multivariate logistic regression models -built by exclusively including those factors testing at the univariate analysis- with subsequent OR calculation; Positive Predictive Value (PPV), Negative Predictive Value (NPV), sensitivity, specificity, Positive Likelihood Ratio (PLR), Negative Likelihood Ratio (NLR) and accuracy were calculated. Statistical significance for sensitivity, specificity, and accuracy was assessed with “exact” Clopper-Pearson test; log method was used for the likelihood ratios; standard logit confidence intervals was used for predictive values. OR, PPV, NPV, sensitivity, specificity, PLR, NLR and accuracy were calculated with and without OLP patients, in order to understand OLP role as bias influencing NBI reliability in detecting HGD/Cis/OSCC.

## Results

Overall clinical-pathological features of patients are summarized in Table 1. One hundred and forty-nine (149) consecutive patients underwent oral cavity inspections in the considered period with NBI/BWL flexible fiberscope. Fifty-one patients were discarded: 18 required biopsy for diagnosis but they refused or withdrew, 33 had non-biopsied FKs not reaching 24-months follow-up. One hundred and six (106) lesions from 98 patients were thus finally considered for this study (median age 62 years – range 19–90; mean age 61 ± 13.7 years). During the period considered for this retrospective study, they underwent visits every 1, 3, 4 or 6 months according their condition/anamnesis, for a total of 556 BWL/NBI inspections, with mean follow-up of 21 ± 13 months. No lesion arisen from specialized epithelium (tongue dorsum) was detected.

**Table 1:** Clinicopathological features of patients

Characteristics	Case no. (%)
Gender	
Male	58 (59.1)
Female	40 (40.9)
Total	98 (100.0)
Age in years (mean ± standard deviation)	61 ± 13.7
Smoking	
Yes	38 (38.7)
No	43 (43.8)
Ex-smoker	17 (17.5)
Presence of removable dentures	
Yes	53 (54.0)
No	45 (46.0)
Anamnesis of radiotherapy	
Yes	5 (5.1)
No	93 (94.9)
Anamnesis of OSCC	
Yes	26 (24.5)
No	80 (75.5)
Type of epithelium	
Tongue epithelium	30 (28.3)
Specialized epithelium	0(0)
Masticatory epithelium	25 (23.6)
Lining epithelium	51 (48.1)
Topographic location of lesions	
Tongue	30 (28.4)
Buccal Mucosa	47 (44.3)
Hard Palate	11 (10.4)
Soft Palate	3 (2.8)
Gum	10 (9.4)
Floor of mouth	5 (4.7)
Total	106 (100.0)
IPCL pattern by NBI	
Pattern I	18 (16.9)
Pattern II	40 (37.7)
Pattern III	24 (22.7)
Pattern IV	24 (22.7)
Diagnoses	
OSCC/Cis	19 (17.9)
HGD	4 (3.8)
PVL	3 (2.8)
LGD	20 (18.9)
OLP	30 (28.3)
FK	30 (28.3)

*OSCC/Cis* oral squamous cell carcinoma/ carcinoma in situ, *HGD* high grade dysplasia, *PVL* proliferative verrucous leukoplakia, *LGD* low grade dysplasia, *OLP* oral lichen planus, *FK* frictional keratosis

Detected lesions included: OSCC/Cis (*n* = 19), HGD (*n* = 4), low grade dysplasia (LGD, *n* = 20), proliferative verrucous leukoplakia (PVL, *n* = 3), OLP (*n* = 30) and FK (*n* = 30). The distribution of statistically significant values according to NBI IPCL pattern -expressed in percentage- is summarized in Table 2. Influence of Head & Neck radiotherapy history was not assessed, as statistical sample was considered inadequate - only 1 patient had undergone such treatment. Age, sex, smoking, use of dentures and anamnesis of OSCC was not found significantly associated to NBI patterns (*p* > 0.5); similarly, no statistical significance was found between use of dentures and FK. On the other hand, site of the lesion and type of epithelium were found significantly associated (*p* = 0.014, *p* = 0.002 respectively) with NBI patterns. Tongue was the anatomical site/type of epithelium most strongly associated with pattern IV (62.5%). Type of lesion was also found significantly associated to NBI pattern (*p* < 0.001), with OSCC/Cis being the lesions most strongly associated to pattern IV. Lesions were then grouped in OSCC/Cis/HGD -with PVL adjunct to this group, due to its high malignant transformation potential [18], non-OSCC/Cis/HGD (benign lesions), non-OSCC/Cis/HGD without OLP (benign lesions without OLP). These groups were found to be statistically significantly associated to NBI patterns (*p* < 0.001): when a pattern IV was shown, histological analysis revealed OSCC/Cis/HGD in 91.7% cases and LGD in 8.3%; pattern III revealed OSCC/Cis/HGD in 12.5% of total cases and in 27.2% when OLP patients were excluded; pattern I and II were strongly associated to benign lesions both with and without OLP patients. Pattern IV was never associated to FK or OLP.

**Table 2:** Prevalence of statistically significant values according to NBI IPCL pattern

Value (statistical significance)	Pattern I(%)	Pattern II(%)	Pattern III(%)	Pattern IV (%)	Total
Type of lesion (*p* < 0.001)					
OSCC/Cis	0	1(2.5)	1(4.2)	17(70.8)	19(17.9)
HGD	0	0	1(4.2)	3(12.5)	4(3.8)
PVL	0	0	1(4.2)	2(8.3)	3(18.9)
LGD	4 (22.2)	7(17.5)	7(29.2)	2(8.3)	20(2.8)
OLP	3 (16.7)	14(35)	13(54.2)	0	30(28.3)
K	11(61.1)	18(45)	1(4.2)	0	30(28.3)
Total	18(100)	40(100)	24(100)	24(100)	106(100)
Malignant lesions vs benign lesions comprehending OLP (*p* < 0.001)					
OSCC/Cis/HGD^a^	0	1(2.5)	3(12.5)	22(91.7)	26(24.5)
Benign lesions	18(100)	39(97.5)	21(87.5)	2(8.3)	80(75.5)
Total	18(100)	40(100)	24(100)	24(100)	106(100)
Malignant lesions vs benign lesions not-comprehending OLP (*p* < 0.001)					
OSCC/Cis/HGD^a^	0	1(3.8)	3(27.2)	22(91.7)	26(34.2)
Benign lesions without OLP	15(100)	25(96.2)	8(72.8)	2(8.3)	50(65.8)
Total	15(100)	26(100)	11(100)	24(100)	76(100)
Site of lesions (*p* = 0.014)					
Tongue	3(16.7)	6(15)	6(25)	15(62.5)	30(28.3)
Buccal mucosa	8(44.4)	23(57.5)	13(54.2)	3(12.5)	47(44.3)
Hard palate	4(22.2)	4(10)	1(4.2)	2(8.3)	11(10.4)
Soft palate	1(5.6)	1(2.5)	0	1(4.2)	3(2.8)
Gum	2(11.1)	5(12.5)	2(8.3)	1(4.2)	10(9.4)
Floor of mouth	0	1(2.5)	2(8.3)	2(8.3)	5(4.7)
Total	18(100)	40(100)	24(100)	24(100)	106(100)
Type of epithelium (*p* = 0.002)					
Tongue	3(16.7)	6(15)	6(25)	15(62.5)	30(28.3)
Masticatory	6(33.3)	11(27.5)	4(16.7)	4(16.7)	25(23.6)
Lining	9(50)	23(57.5)	14(58.3)	5(20.8)	51(48.1)
Total	18(100)	40(100)	24(100)	24(100)	106(100)

*OSCC/Cis* oral squamous cell carcinoma/ carcinoma in situ, *HGD* high grade dysplasia, *PVL* proliferative verrucous leukoplakia, *LGD* low grade dysplasia, *OLP* oral lichen planus, *FK* Frictional Keratosis

^a^PVL was adjunct to this group due to its high malignant transformation potential

Our data thus revealed a significant association only of pattern IV with the OSCC/Cis/HGD group, but literature usually group pattern III and IV [10, 12, 13] to evaluate NBI efficacy in detecting OSCC/Cis/HGD; thus multivariate analysis/OR, PPV, NPV, sensitivity, specificity, PLR, NLR and accuracy were calculated with and without OLP patients for pattern IV alone and for aggregate pattern III and IV. Results are summarized in Tables 3 and 4.

**Table 3:** Evaluation of NBI pattern III-IV and pattern IV alone as a diagnostic test for OSCC/Cis/HGD, with and without OLP patients

Diagnostic test	Pattern III-IV (95% CI)	Pattern IV (95% CI)
Sensitivity		
With OLP patients	96.2% (80.4–99.9%)	84.6% (65.1–95.6%
Without OLP patients	96.2% (80.4–99.9%)	84.6% (65.1 95.6%)
Specificity		
With OLP patients	71.3% (60.0–80.8%)	97.5% (91.2–99.7%)
Without OLP patients	80.0% (66.3–89.9%)	96.0% (86.3–99.5%)
PLR		
With OLP patients	3.34 (2.35–4.76)	33.85 (8.53–134.30)
Without OLP patients	4.81 (2.75–8.41)	21.15 (5.39–83.06)
NLR		
With OLP patients	0.05 (0.01–0.37)	0.16 (0.06–0.39)
Without OLP patients	0.05 (0.01–0.33)	0.16 (0.06–0.40)
PPV		
With OLP patients	52.1% (43.3–60.7%)	91.7% (73.5–97.7%
Without OLP patients	71.4% (58.8–81.4%)	91.7% (73.7–97.7%)
NPV		
With OLP patients	98.3% (89.2–99.7%	95.1% (88.8–97.9%
Without OLP patients	97.6% (85.3–99.6%)	92.3% (82.9–96.7%)
Accuracy		
With OLP patients	77.4% (68.2–84.9%)	94.3% (88.1–97.9%)
Without OLP patients	85.5% (75.6–92.5%)	92.1% (83.6–97.1%)

*PLR* positive likelihood ratio, *NLR* negative likelihood ratio, *PPV* positive predictive value, *NPV* negative predictive value, *CI* confidence interval

**Table 4:** Multivariate analysis, adjusted for terms of gender, age, smoking habits, of NBI patterns related to histopathologic diagnosis

Non- HGD/Cis/OSCC	HGD/Cis/OSCC detected by NBI pattern III-IV	HGD/Cis/OSCC detected by NBI pattern IV
With OLP patients	OR:79.04 *(95%CI:9.5–652.4)*	OR:261.7 *(95%CI:37.7–1815.5)*
Without OLP patients	OR:103.1 *(95%CI:19.8–897.4)*	OR:161.7 *(95%CI:22.1–1180.2)*
OLP patient only	OR: not evaluable	OR:53.1 *(95%CI:5.06–556.1)*

*OSCC/Cis* oral squamous cell carcinoma/ carcinoma in situ, *HGD* high grade dysplasia, *OR* odds ratio, *CI* confidence interval

Multivariate analysis/OR was also calculated for a Non-OSCC/Cis/HGD population of OLP patients only; the assessment was possible for pattern IV only, as no OLP showed NBI pattern III. All these statistical evaluations were found to be significant (*p* < 0.05/CI = 95%). The presence of OLP patients in our cohort did not influence sensitivity and NLR both for pattern III and IV grouped and pattern IV alone (96.2%-0.05 and 84.6%-0.16, respectively). Pattern III and IV grouped showed the highest sensitivity in the search of OSCC/Cis/HGD but also the lowest specificity, both with and without OLP patients (71.3 and 80.0%, respectively), due to a high number of false positive. The best value of specificity was reached when the pattern IV was considered singularly associated to OSCC/Cis/HGD, higher in presence rather than in absence of OLP patients (97.5 and 96.0%, respectively) due to a higher number of true negative. PPV was dramatically increased by considering pattern IV alone (91.7%), and did not suffer of any reduction by the presence of OLP patients. PPV of pattern III and IV grouped was low (more numerous false positive cases), remarkably reduced in the OLP cohort (from 71.4 to 52.1%). Similar values of NPV were reached between pattern IV alone and pattern III and IV grouped; presence of OLP patients affected NPV slightly (from 98.3 to 97.6% for pattern III-IV, from 95.1 to 92.3% for pattern IV). Finally, global accuracy was higher when pattern IV was considered alone, minimally influenced by the presence/absence of OLP patients (94.3 and 92.1%, respectively). Presence of OLP patients in the cohort did reduce the significance (OR) at multivariate analysis for pattern III-IV, while increased significance of pattern IV alone (OR: 261.7; CI 37.7–1815.5). Multivariate analysis also showed significance for pattern IV when the cohort included OSCC/Cis/HGD and OLP patients only.

**Fig. 2:**
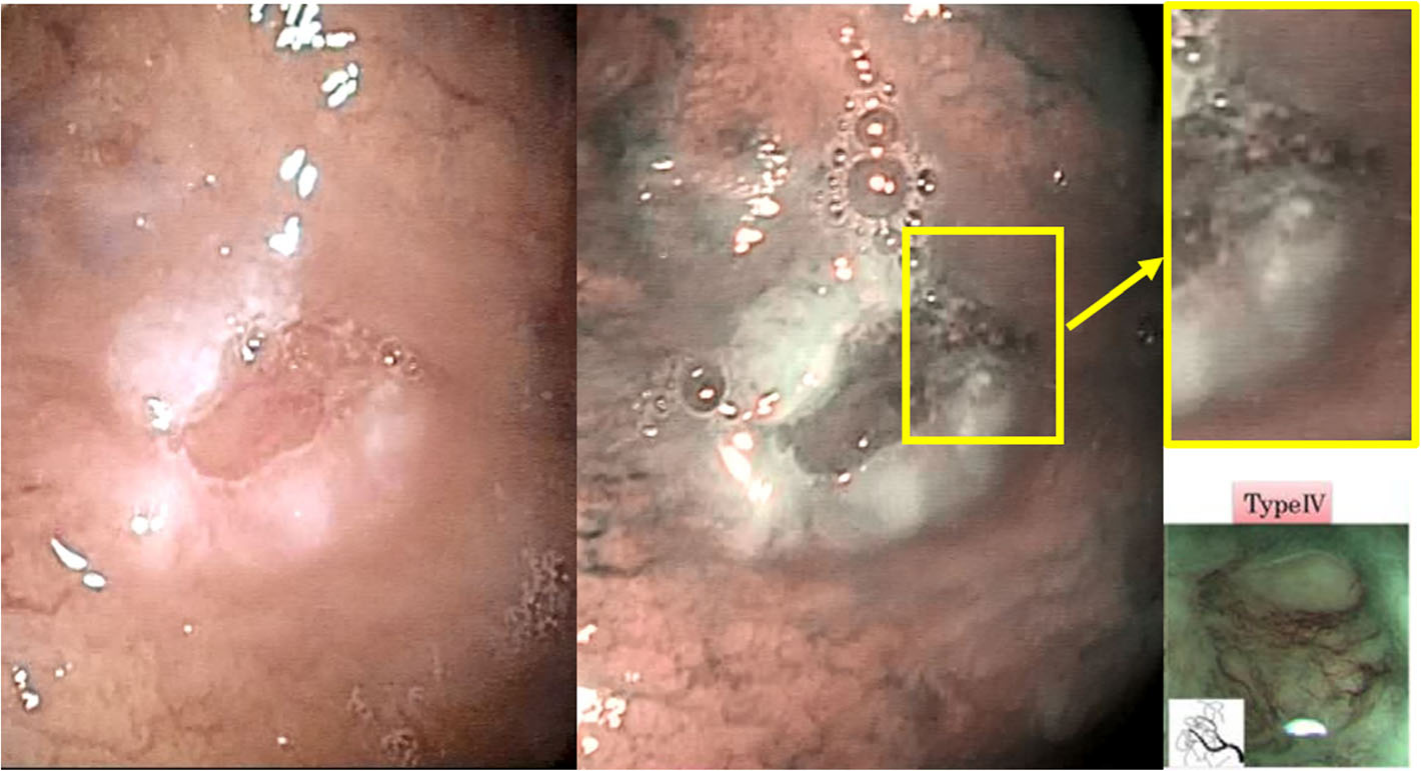
Early diagnosis of OSCC recurrence through NBI. The inspection of this asymptomatic surgical scar (outcome of a second-intention healing), from a patient who had previously had surgery for OSCC, did not reveal suspicious areas – such as ulcers, lumps, red and/or white lesions - at BWL (left image). Still, it was biopsied due to an anomalous vascularization at NBI visualization (highlighted spot, middle image) which was classified as IPCL pattern IV (upper and lower [2] - right images). Histopathological examination showed a recurrence of OSCC

**Fig. 3:**
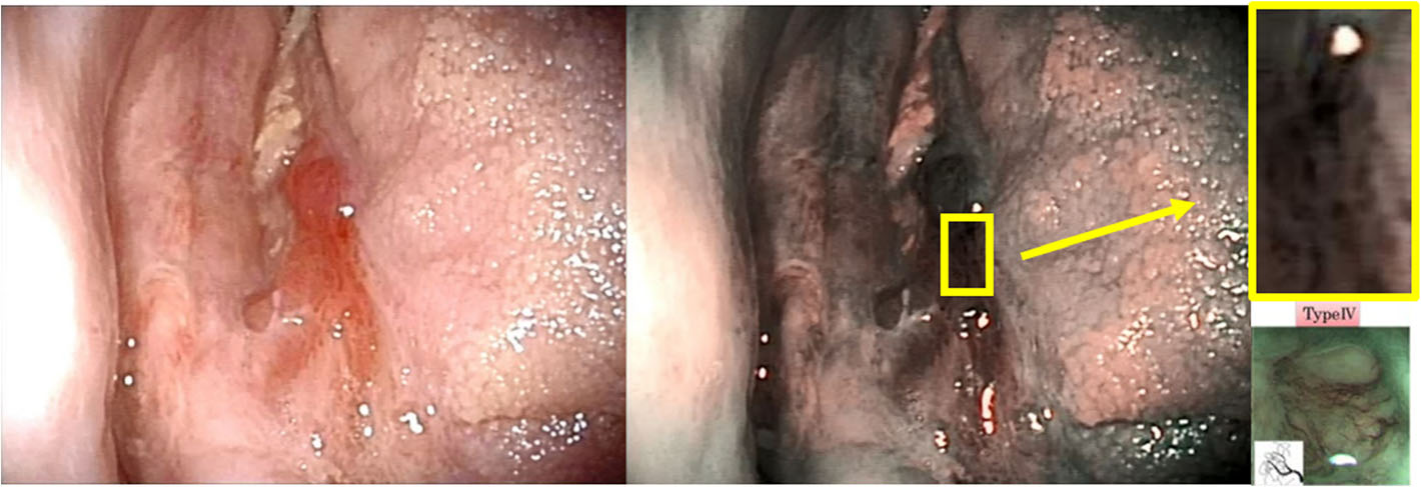
Early diagnosis of OSCC in OPMD patients through NBI. In a patient with multiple ulcers and erosions due to erosive LP, a biopsy was performed on this ulcer (left image) among the various lesions, as it showed an anomalous vascularization at NBI visualization (highlighted spot, middle image), which was classified as IPCL pattern IV (upper and lower [2] - right images). Histopathological examination confirmed that an occurred malignant transformation (OSCC) was intercepted

With descriptive purposes, we report that in one patient with no denture, a lesion from buccal mucosa of the posterior cheek with clinical characteristics (linear white patch, homogeneous, not augmented in consistency) of FK showed NBI pattern III; despite selective grinding of sharp edges of neighboring teeth, the lesion showed the same characteristics after 2 weeks. After obtaining informed consent from the patient, the lesion was biopsied and its non-dysplastic nature was confirmed histologically; the lesion has been thus inserted in the FK group. On the other hand, in two patients being followed-up for previously treated OSCC, the inspection showed no areas with classical suspicious clinical aspect at BWL but one pattern IV NBI spot was revealed (Fig. 2). Local biopsies were performed, after informed consent was obtained, and in both cases OSCC recurrence was early diagnosed. In another patient, in follow-up for an erosive LP, which had steadily shown NBI patterns II – III for 16 months, OSCC was early diagnosed with a biopsy performed consequently to a NBI pattern switch to IV (Fig. 3).

## Discussion

Several authors [20] have highlighted that OSCC remains a lethal disease in more than 50% of cases; such low rates of survival are mainly linked to the fact that most cases are diagnosed in advanced stages, despite accessibility of the mouth for regular examination. Thus, the common objective for both patients and physicians must be early detection. Specialists need to be trained to detect early signs/symptoms in order to facilitate treatment, increasing effectiveness and reducing morbidity [21, 22]. Clinical features of OSCC include:

- Red, white, mixed red/white (speckled) or irregular-white lesion (erythroplakia, leukoplakia, erythroleukoplakia and verrucous leukoplakia, respectively)
- Ulcer, eventually with fissuring or raised exophytic margins
- Mass, with or without ulcerations
- Pain/ loss of sensitivity (numbness) to any region of the lower third of the face
- Tooth/teeth with high-grade mobility or extraction socket not healing after tooth/teeth extraction(s)

As apparent, clinicians’ view of the lesion may be biased by multiple factors when relying to these criteria only [21–23]. Among the numerous attempts in searching for help to overcome limits of clinical criteria, including vital staining [24] and other optical techniques [25–27], literature has shown that NBI endoscopy has a great potential of improving clinicians’ possibility of performing early diagnosis. Despite excellent results, its routinary use in the inspection of oral cavity may present some difficulties at the present stage of scientific literature. History of head & neck radiotherapy is considered to be a major bias in the NBI visualization of the larynx/pharynx [17], but there are no data about oral cavity. In our retrospective study, unfortunately, we were unable to evaluate the influence of Head & Neck radiotherapy on NBI patterns, due to an insufficient sample. On the other hand, smoking habit and presence of dentures were not found statistically associated to NBI patterns. Another major bias in the NBI visualization of the larynx/pharynx is the site of the lesion, due to different epithelium thickness from site to site, but literature is not concordant on this aspect [10, 11] in oral cavity. In our study, anatomical site and type of epithelium were assessed as statistically significant, with most of NBI pattern IV located on the keratinized epithelium of tongue lateral borders. Furthermore, we found that the distribution of NBI patterns in the thin non-keratinized lining epithelium was similar to the thicker masticatory epithelium. Thus in our study thicker epithelium does not seem to hide IPCLs, but wider studies are needed to understand correlation between pattern IV and tongue lateral borders, maybe influenced but the higher rate of OSCCs compared to other sites [16].

NBI has proved remarkably positive results: when IPCL pattern III and IV are detected, literature showed a high correlation to OSCC/Cis/HGD [9, 10, 12, 13]. Yet, studies generally exclude OPMDs, especially OLP, as they are considered major bias [9–11]. These lesions often have dishomogeneous/ulcerated aspects and show high-grade NBI patterns. In our retrospective study, we assessed NBI efficacy in routine clinical use, on a cohort of patients affected by oral lesions, not normalized for possible bias suggested by present literature. This led to differences respect to data shown by literature, in which the specificity is usually higher than the sensitivity and, in general, oral cavity lesions are often grouped with oropharynx lesions and sometimes with laryngeal/nasopharyngeal lesions too [9, 10]. In order to acknowledge the OLP influence as a possible bias, we performed every statistical calculation with and without OLP patients. Our results showed that the presence of OLP patients in our cohort did not influence sensitivity and NLR. This means that, in terms of routine use of NBI, the possibility that a patient has OLP does not impair NBI reliability in detecting lesions positive for OSCC/Cis/HGD; our results show that both pattern III and IV (highest sensitivity when grouped) should be taken into account for biopsy, as advised by literature. On the other hand, OLP may lead to false positive cases when considering pattern III and IV as at risk (low specificity due to high rates of OLP showing pattern III), but it does not seem to impair pattern IV reliability, as confirmed by multivariate analysis. This could pave the way to future perspectives, meaning that patients with multiple/single OLP lesion(s), after initial diagnostic biopsy, could be followed up with NBI, performing a new biopsy (in addition to cases in which clinical appearance changes/becomes suspicious) when pattern IV is detected, as a sign of occurred malignant transformation - as happened once in our cohort of patients. Furthermore, both global accuracy and OR reached best results when pattern IV was considered alone; further studies are needed with the aim to interpret such result, which could highlight pattern IV as a clinical marker of OSCC/Cis/HGD presence. Another possible factor that influenced statistical outcome is the wide sample of FK. The diagnosis of FK is considered to be clinical and, being completely benign lesions, no treatment is required [19]. Even if there is no evidence that minor continuative trauma has carcinogenic potential, the removal of irritant factors is advised but sometimes not possible (e.g.: teeth malposition/absence). Yet, it is a common possibility that a patient may be affected by multiple/single FK(s), leaving the clinician with doubts about the necessity of a biopsy, especially in presence of risk factors (e.g.: smoking habit). Data from our study show that FKs are associated with NBI pattern I and II; this could be of great help for the clinician in the follow-up of patients with single/multiple FK(s), especially in presence of risk factors (e.g.: smoking habit, which from our study does not seem to alter NBI inspection result).

As a matter of fact, the present study has some limitations which need to be underlined. First, in order to perform a diagnosis of OLP, just a small part of the lesion(s) was (were) excised, and this may have resulted in sampling mistakes. Second, this study is retrospective on a relatively small cohort of patients. Bigger prospective multicentric studies should be performed to confirm obtained data. Another limitation that needs to be stressed is the lack of a widely accepted definition of “expert” and “experienced” in NBI procedures – especially in the field of oral cavity. This is a considerable criticism of present NBI literature [9] and potentially a major bias. In our experience it may require a period of several months/1 year to became expert. Classical gastrointestinal endoscopy criteria [28] state that at least 130–140 endoscopic evaluations are necessary to achieve competence. In our case, the operator (AG) had performed at least 140 procedures (NBI oral exams) per year for 5 years. General consensus should be achieved to define the status of “experienced” and “expert”. Possibly, application to NBI of classical gastrointestinal endoscopy criteria of 130–140 procedures to set the status of “experienced” operator could be suitable, but further studies on operators’ learning curve are needed to avoid the risk of major bias when evaluating NBI reliability. Furthermore, as diagnostic percentage standards are extensively shown in literature, self-evaluation, comparing operator’s own NBI pattern evaluation with histopathological analysis, could be easily achievable.

## Conclusion

- NBI has showed high sensitivity and NPV in detection of OSCC/Cis/HGD also in a cohort of patients with oral cavity lesions non-normalized for possible confounding factors, confirming its high impact in anticipating first diagnosis of malignancies.
- Presence of OLP patients in a cohort do not lead to false negatives but may raise false positive rate; this must be kept in mind e.g. during follow-up.
- NBI could help in the follow-up of patients with multiple chronic lesions, both in case of no need for treatment (e.g. FK) both for help the diagnosis of occurred malignant transformation (e.g. OLP) through detection of IPCL pattern IV;
- IPCL pattern IV does seem to be the most significantly associated to OSCC/Cis/HGD, as confirmed by the highest value of accuracy and OR;
- The results obtained from this study may function as a pilot for prospective multicentric studies with bigger samples, to confirm such findings.

## Abbreviations

BWL: Broad white light
CI: Confidence interval
Cis: Carcinoma in situ
FK: Frictional keratosis
HGD: High-grade dysplasia
HNSCC: Head & neck squamous cell carcinoma
IPCL: Intrapapillary capillary loops
LGD: Low-grade dysplasia
NBI: Narrow band imaging
NCCN: National cancer comprehensive network
NLR: Negative likelihood ratio
NPV: Negative predictive value
OLP: Oral lichen planus
OPMD: Oral potentially malignant disorder
OR: Odds ratio
OSCC: Oral squamous cell carcinoma
PLR: Positive likelihood ratio
PPV: Positive predictive value
SPSS: Statistical package for social science
WHO: World health organization
